# Status and influencing factors of spiritual climate among oncology nurses: a cross-sectional study

**DOI:** 10.3389/fpsyg.2026.1759724

**Published:** 2026-05-14

**Authors:** Tianyue Zhang, Chunfeng Dong, Jie Tang, Ping Yu

**Affiliations:** 1 Wuxi School of Medicine, Jiangnan University, Binhu, Wuxi, Jiangsu, China; 2 Jiangnan University Medical Center (Wuxi No.2 People's Hospital), Liangxi, Wuxi, Jiangsu, China

**Keywords:** cross-sectional study, influencing factors, oncology nurse, spiritual climate, status

## Abstract

**Background:**

Oncology nurses frequently experience significant physical and psychological stress. These negative emotions may adversely affect the collective spiritual climate within the department, leading to professional burnout, which in turn impacts the stability of the nursing team and the quality of care delivered. This study aims to assess the spiritual climate among oncology nurses and identify its potential associated factors.

**Methods:**

A cross-sectional study was conducted among 321 oncology nurses from 43 hospitals across 13 cities in Jiangsu Province, China, using convenience sampling between April and May 2025. Data were collected through the General Information Questionnaire, Spiritual Climate Scale (SCS), Perceived Organizational Support Scale (POS), Occupational Coping Self-Efficacy Scale, and Inclusive Leadership Scale (ILS). Statistical analysis was performed using SPSS 27.0.

**Results:**

In total, 321 participants completed the survey. After data cleaning, 273 were valid and included in the final analysis. The Spiritual Climate Scale score among oncology nurses was 81.11 ± 15.04. There were significant differences in Spiritual Climate Scale score according to age (*F* = 2.780, *p* = 0.042), administrative position (*F* = 3.828, *p* = 0.023) and the number of night shifts per month (*F* = 3.210, *p* = 0.024). Multiple linear regression analysis identified head nurses, perceived organizational support, occupational coping self-efficacy, and inclusive leadership as factors significantly associated with spiritual climate (*p* < 0.05).

**Conclusions:**

The findings suggest oncology nurses generally perceive a positive spiritual climate, though improvements are still needed. Nursing managers should enhance spiritual climate by adopting targeted interventions based on the factors influencing them.

## 1 Introduction

As an indispensable force in the healthcare team, nurses play an important role in safeguarding public health, ensuring patient safety and promoting the development of healthcare (Wang et al., 2024). According to the 2022 Global Cancer Statistics, there were approximately 20 million new cancer cases and 9.7 million cancer deaths worldwide (Bray et al., 2024). As frontline healthcare providers with the most frequent patient contact, oncology nurses face multiple challenges including staffing shortages, heavy workloads, and occupational exposure to chemotherapeutic agents. Moreover, they must

continually confront emotionally distressing situations involving patient suffering and death. Prolonged exposure to such demanding work environments predisposes them to professional burnout and other negative psychological states, which may ultimately lead to existential doubts regarding their professional purpose (Shanafelt, 2009). This concerning situation adversely affects nurses' physical and psychological well-being as well as their career progression, while concurrently compromising the quality of patient care delivery (Xu, 2024). (Çinar Özbay et al. 2025) found that pediatric oncology nurses recognize the impact of trauma but lack sufficient organizational support. This gap between emotional needs and workplace support accumulates over time, eroding nurses' psychological resources and their capacity to find meaning in work. A supportive environment providing emotional safety and opportunities for meaning-making can help bridge this gap, enabling nurses to better process traumatic experiences and maintain well-being.

The concept of “spirituality” in healthcare has gained significant scholarly attention in recent years, with numerous researchers emphasizing its critical role in holistic patient care (Ílvia Caldeira and Timmins, 2017). The notion of a spiritual climate refers to the shared perceptions of employees toward spirituality and both the state and process of sharing workplace spirituality (Cruz et al., 2018). It encompasses both the existing state of workplace spirituality and the dynamic process of sharing spiritual values among colleagues. More specifically, it reflects the extent to which the work environment supports spiritual expression through its practices, communication, and systems, fostering collaboration centered on meaning, purpose, and a sense of connectedness (Cruz et al., 2022). (Doram et al. 2017) in the United States defined spiritual climate as an organizational environment that fosters respect for individual expression and encourages employees to voice their perspectives. The research team had developed the Spiritual Climate Scale (SCS) for the Healthcare Industry, a validated instrument designed to assess healthcare professionals' perceptions of their work environment's spiritual climate. Research (Cruz et al., 2020) demonstrated that nurses are more likely to find meaning in their work when employed in a positive workplace spiritual environment. Such settings enable nurses to provide comprehensive humanistic care and promote the development of the unit with a collective interest in mind. Jiangsu Province, a medical hub in coastal China, reflects typical nursing patterns in Chinese public hospitals. Oncology nurses manage chemotherapy, symptom control, palliative care, and psychosocial support. The demanding nature of oncology nursing, with high patient turnover and low nurse-to-patient ratios, shapes a distinct work environment that influences nurses' spiritual climate perceptions.

Social Cognitive Theory (SCT) posits that there is an interactive relationship among individual perception, behavior, and the environment (Chen and Tu, 2021). As an essential personal factor, occupational coping self-efficacy plays a vital role in developing an individual's occupational health (Lin et al., 2024). Meanwhile, both perceived organizational support and leadership function as significant environmental factors (Gillet et al., 2013; Huang et al., 2025). Nurses' occupational coping self-efficacy is more concerned with exploring how confident nurses are in their ability to deal with the demands of the job (Mei and Han, 2021). High professional self-efficacy, by reducing emotional exhaustion (Lin et al., 2024), may empower employees to engage in more authentic communication and collective meaning-building, thereby fostering their perception of a more positive spiritual climate.

Perceived organizational support refers to an employee's subjective belief that the organization values their contributions and cares about their well-being (Rhoades and Eisenberger, 2002). It has been shown (Huang et al., 2021) the perceived organizational support positively predicts nurses' perceived spiritual climate. Perceived organizational support not only provides nurses with a sense of recognition and belonging but also ensures access to necessary resources, including those for professional development. Meeting nurses' educational needs through organizational investment (Kudubeş et al., 2023) further enhances their competence and confidence. Therefore, perceived organizational support may serve as a significant environmental factor influencing spiritual climate among oncology nurses.

(Doram et al. 2017) and (Cruz et al. 2018) suggest creating a positive workplace spiritual environment requires managers to be open and actively listen and respond to the feelings and views of nurses. In other words, leadership can be a driver of an increased spiritual climate. Inclusive leadership is characterized by openness, accessibility and availability (Carmeli et al., 2010). Leaders with this style respect individual diversity, recognize individual value of employees, encourage collaboration and communication, tolerate team members' failures and provide corresponding professional guidance. Accordingly, it may also be one of the important environmental factors influencing spiritual climate among oncology nurses.

**Figure 1:**
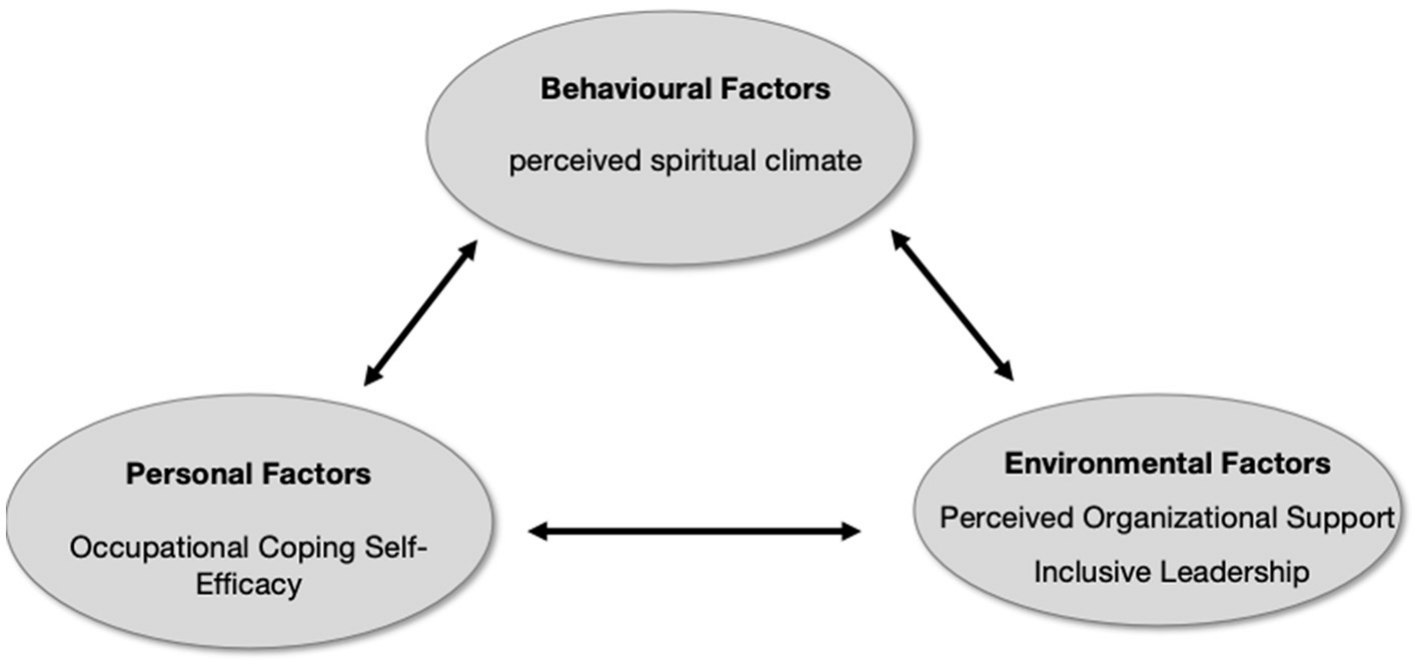
A conceptual model of the hypothetical relationship between spiritual climate and related factors in oncology nurses.

While scholarly attention to nurses' perceptions of workplace spiritual climate has grown significantly, research specifically targeting oncology nurses remains relatively limited and insufficiently comprehensive. To address this gap, this study developed a conceptual model based on Social Cognitive Theory. As shown in Figure 1, we hypothesize that occupational coping self-efficacy, organizational support and inclusive leadership are important influencing factors for spiritual climate among oncology nurses.

## 2 Methods and materials

### 2.1 Study design and participants

A convenience sampling method was employed to recruit oncology nurses from 43 hospitals across 13 cities in Jiangsu Province between April and May 2025. The 43 hospitals were selected based on their geographic distribution and their willingness to participate. The participating hospitals included 31 tertiary hospitals (72.1%) and 12 secondary hospitals (27.9%). In terms of hospital type, five were specialized oncology hospitals (11.6%) and 38 were general hospitals with oncology departments (88.4%). The inclusion criteria were as follows: (1) clinical nurse staff who were hospital employees possessing a certificate of nursing practice from the People's Republic of China; (2) nurses who had worked in the oncology department for 1 year or longer; (3) nurses who agreed to participate voluntarily in this study. The exclusion criteria were (1) nurses on maternity leave, sick leave or separation status during the survey period; (2) nurses undergoing advanced training.

*A priori* power analysis was performed using the G^*^power 3.1.9.7 software (Faul et al., 2009), where the required sample size was calculated by using *F*-tests and linear multiple regression: fixed model, R^2^ deviation from zero as the statistical test, and “*A priori*: compute required sample size—given α, power, and effect size” as the type of power analysis. Cohen's *f*^2^ = 0.15 with medium effect size, α = 0.05, power (1 – β) = 0.80, and 9 predictors were taken in the model. The result of the analysis indicated that the minimum required sample size was 137. Meanwhile, considering 20% sample size loss, we aimed to recruit at least 172 participants. The number of independent variable was predetermined based on our theoretical framework and review of relevant literature (Guo et al., 2023). We ultimately obtained a final sample of 321 oncology nurses, which provided sufficient statistical power for the analysis.

### 2.2 Measurements

The instruments were selected based on Bandura's Social Cognitive Theory, which posits that human functioning results from the reciprocal interaction of personal factors, environmental factors, and behavioral factors. Within this framework, the Spiritual Climate Scale (SCS) measures the behavioral outcome, reflecting nurses' shared perceptions of workplace spirituality. The Occupational Coping Self-Efficacy Scale for Nurses (OCSE-N) assesses the personal factor of self-efficacy, capturing nurses' confidence in managing job demands. The Perceived Organizational Support Scale (POS) and the Inclusive Leadership Scale (ILS) capture key environmental factors, namely organizational support and leadership, which shape the workplace context and influence nurses' perceptions. All four scales are well-established instruments with demonstrated reliability and validity in previous nursing research.

#### 2.2.1 General information questionnaire

The research team designed the questionnaire based on a literature review, including 15 items on gender, age, marital status, children's status, years of specialized work, education, professional rank, administrative position, nursing capacity, income, number of night shifts, etc.

#### 2.2.2 Spiritual climate scale (SCS-C)

This study employed the Spiritual Climate Scale (SCS) developed by (Doram et al. 2017). We used its validated full Chinese version (SCS-C) (Wu et al., 2019), comprising four items (1 is “My unit encourages and supports nurses to express their ideas and opinions, and colleagues are able to listen and accept them”, 2 is “Doctors and nurses respect the opinions and ideas that I put forward”, 3 is “I feel a sense of belonging and identity when I communicate and share my ideas with my colleagues”, and item 4 is “I am in a department where everyone can express his or her ideas and we respect and understand each other”). This version underwent cross-cultural adaptation via a standardized translation-back-translation procedure and demonstrated sound reliability and validity among Chinese healthcare professionals. The questionnaire is scored on a 5-point Likert scale, from “strongly disagree” to “strongly agree”. The final score is the average of the four items minus 1, then multiplied by 25, which was distributed in the range of 0–100 points theoretically. The higher the total score, the better the spiritual climate is perceived by the participants. We secured the necessary permissions for its use from the original author. The Cronbach's α of the scale was 0.833 and has good discriminant validity. It is 0.924 in this study. This scale has been authorized by the original author for English translation.

#### 2.2.3 Occupational coping self-efficacy scale for nurses (OCSE-N)

This scale was translated and culturally adapted into Chinese by (Zhai et al. 2021), consisting of two dimensions, namely, occupational burden (four items) and relationship difficulties (five items), with a total of nine items. Each entry is rated on a 5-point Likert scale, with scores ranging from 1 to 5 theoretically, from “unable to cope easily” to “completely able to cope easily”. The higher the score, the higher the level of self-efficacy in occupational coping of the respondents. The Cronbach's α of this scale was 0.807. It is 0.909 in this study.

#### 2.2.4 Perceived organizational support scale (POS)

This scale, adapted by (Zuo and Yang 2009), is widely used in nurses' groups to assess perceived organizational support from the hospital. There are 13 entries, each of which is scored on a 5-point Likert scale, with higher aggregate scores reflecting stronger perceptions of organizational support from the hospital. The Cronbach's α for the two dimensions was 0.847 and 0.904, respectively. In the current study, the overall scale's Cronbach's α is 0.974.

#### 2.2.5 Inclusive leadership scale (ILS)

The scale was developed by (Carmeli et al. 2010). It contains 9 entries in 3 dimensions: openness, availability and accessibility. Each entry is rated on a 5-point Likert scale, with higher scores indicating better inclusive leadership behaviors as perceived by nurses. The ILS has well documented psychometric properties, its Cronbach's α was 0.940. In this study it is 0.969.

### 2.3 Data collection

The researchers distributed questionnaires (electronic version) via “Questionnaire Star” platform online. After obtaining permission from the nurse administrators at the identified hospitals, researchers directly distributed the online questionnaire links to nurses during their free time. Before distribution, the purpose, significance, content, precautions, and the principle of anonymity and voluntary participation were explained. The survey could only proceed after participants had given their consent. And participants could withdraw at any time. Each *IP*/account could submit the questionnaire only once. A rigorous data cleaning protocol was applied: (1) An attention-check item (“Please select “disagree” for this item to verify your attention”) was embedded within the questionnaire; (2) Questionnaires completed in less than 1 min or more than 10 min were excluded.

### 2.4 Statistical analysis

Statistical analysis was completed using the Statistical Package for SPSS software version 27.0. Categorical data are presented as frequency and constituent ratio, while normally distributed continuous data are described using mean ± standard deviation. *Pearson* correlation analysis was used to analyze the relationship between spiritual climate and related variables. A multivariate linear regression analysis was conducted to explore the influence of various factors on spiritual climate. *p* < 0.05 was considered statistically significant. Independent variables were selected based on Social Cognitive Theory and literature review. Occupational coping self-efficacy was included as a personal factor, while perceived organizational support and inclusive leadership were included as environmental factors. Demographic variables that were significant in univariate analyses were also entered as independent variables.

### 2.5 Validity and reliability of instruments

Confirmatory factor analysis was conducted using Stata 17.0 to examine construct validity. The four-factor model demonstrated a marginally acceptable fit: χ^2^/df = 3.612, CFI = 0.867, RMSEA = 0.098, SRMR = 0.048. Although CFI was slightly below 0.90, the SRMR (0.048) was well below 0.08, indicating acceptable absolute fit (Hu and Bentler, 1999). The suboptimal fit may be attributed to model complexity and relatively high correlations among latent variables, which are theoretically expected. All factor loadings exceeded 0.50 (*p* < 0.001). AVE values ranged from 0.55 to 0.78 (>0.50), and CR values ranged from 0.92 to 0.97 (>0.70), as shown in Supplementary Materials. Discriminant validity was established using the Fornell–Larcker criterion: the square root of the AVE for each construct was greater than its correlations with other constructs, indicating adequate discriminant validity. Given the study's focus on hypothesis testing rather than scale validation, and the instruments' strong reliability, the factor structure was deemed acceptable.

### 2.6 Ethical considerations

This study was carried out in accordance with the Declaration of Helsinki. Ethical approval was obtained from the Ethics Committee of Wuxi No.2 People's Hospital (ethics no. 2025 Y-74). Written informed consent was obtained. Participants' identifying information was set to be anonymized in order to protect their personal information.

## 3 Results

**Figure 2:**
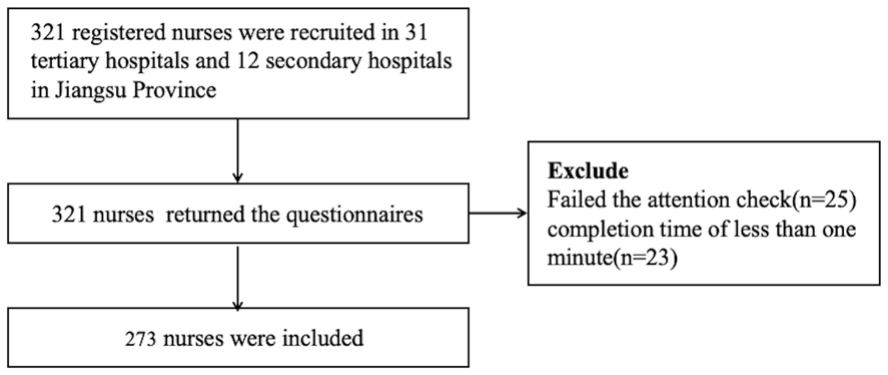
Flow charts on inclusion of studies.

A total of 321 questionnaires were distributed, and all were returned. After data cleaning, 48 invalid responses were excluded, including 25 that failed the attention check and 23 with abnormally short completion times (less than 1 min). This resulted in a final analytic sample of 273 participants (85.0% valid response rate). The flowchart is shown in Figure 2. The 273 participants were distributed across 43 hospitals.

### 3.1 Common method bias test

Common method bias was assessed using Harman's single-factor test. An exploratory factor analysis including all measurement items revealed 10 factors with eigenvalues greater than 1. The first factor accounted for 36.52% of the total variance, which was below the recommended threshold of 40%, suggesting that common method bias was not a significant concern in this study.

### 3.2 Demographic factors influencing spiritual climate among oncology nurses

A total of 273 oncology nurses completed the survey. There was a significant difference in perceived spiritual climate among oncology nurses of different age, administrative position and the number of night shifts per month (*p* < 0.05), as shown in Table 1. *Post hoc* comparisons showed that oncology nurses under 25 years old had significantly higher SCS scores than the 25–35 nurses (*p* = 0.023) and the over 45 nurses (*p* = 0.015); Head nurses had significantly higher SCS scores than nurses without administrative position (*p* = 0.018); Nurses working no night shifts monthly had significantly higher SCS scores than those working more than nine night shifts per month (*p* = 0.021).

**Table 1:** Sociodemographic characteristics of oncology nurses and the scores of SCS (*N* = 273).

Item		*N* (%)	SCS total scores ($\bar{x}\pm$s)	*t/F*	*p*
Gender	Male	9 (3.3)	81.94 ± 24.09	0.168	0.866
	Female	264 (96.7)	80.08 ± 14.71		
Age	< 25	30 (11.0)	86.67 ± 13.31	2.780	0.042
	25–35	145 (53.1)	79.83 ± 15.95		
	36–45	72 (26.4)	82.90 ± 13.56		
	>45	26 (9.5)	76.92 ± 14.01		
Marriage	Married	195 (71.4)	81.25 ± 15.11	0.276	0.759
	Unmarried	74 (27.1)	80.49 ± 15.10		
	Divorced	4 (1.5)	85.94 ± 12.88		
Children	No children	97 (35.5)	80.67 ± 14.88	0.449	0.639
	1	131 (48.0)	80.77 ± 15.69		
	>1	45 (16.5)	83.06 ± 13.56		
Educational	Associate degree	39 (14.3)	80.12 ± 16.34	0.115	0.891
	Bachelor's degree	229 (83.9)	81.30 ± 14.90		
	Master's degree	5 (1.8)	80.00 ± 13.55		
Professional rank	Nurse	37 (13.6)	81.93 ± 18.27	0.098	0.983
	Registered nurse	93 (34.1)	80.78 ± 14.81		
	Senior nurse	94 (34.4)	80.72 ± 14.94		
	Associate chief nurse	42 (15.4)	81.70 ± 13.69		
	Chief nurse	7 (2.6)	83.04 ± 11.81		
Administrative position	No position	210 (76.9)	79.79 ± 15.46	3.828	0.023
	Nursing team leader	36 (13.2)	84.38 ± 11.91		
	Head nurse	27 (9.9)	87.04 ± 13.75		
Level of hospital	Secondary hospital	47 (17.2)	80.98 ± 14.86	−0.064	0.949
	Tertiary hospitals	226 (82.8)	81.14 ± 15.11		
Nature of hospital	General hospital	251 (91.9)	81.18 ± 15.40	0.320	0.751
	Specialized oncology hospital	22 (8.1)	80.40 ± 10.44		
Nursing capacity	N1	52 (19.0)	81.13 ± 16.40	0.338	0.852
	N2	119 (43.6)	80.09 ± 15.71		
	N3	73 (26.7)	81.93 ± 13.52		
	N4	18 (6.6)	83.68 ± 13.24		
	Other	11 (4.0)	82.39 ± 15.00		
Years of specialized work	1–3	77 (28.2)	82.14 ± 15.53	0.580	0.629
	4–6	52 (19.0)	81.49 ± 13.78		
	7–9	30 (11.0)	77.92 ± 19.88		
	>9	114 (41.8)	81.09 ± 13.85		
Employment type	Regularly on board	60 (21.0)	80.31 ± 13.66	0.115	0.892
	Contractual	177 (64.8)	81.39 ± 15.21		
	Personnel agency	36 (13.2)	81.08 ± 16.74		
Monthly salary	< 5,000	57 (20.9)	78.73 ± 18.78	1.269	0.283
	5,000–8,000	131 (48.0)	82.44 ± 13.34		
	>8,000	85 (31.1)	80.66 ± 14.68		
Number of night shifts per month	0	66 (24.2)	80.87 ± 15.77	3.210	0.024
	1–4	107 (39.2)	83.06 ± 14.39		
	5–9	76 (27.8)	81.25 ± 14.40		
	>9	24 (8.8)	72.66 ± 15.83		
Religious belief	Yes	17 (6.2)	80.51 ± 16.07	−0.169	0.866
	No	256 (93.8)	81.15 ± 15.01		

### 3.3 Oncology nurses' scores on spiritual climate

As shown in Table 2, the total score for the oncology nurses' spiritual climate was 81.11 ± 15.04. Specifically, item 4 obtained the highest score, while item 2 had the lowest.

**Table 2:** Total score and average scores of each item on the spiritual climate scale (*N* = 273).

Variable	Score ($\bar{x}\pm$s)
SCS-C total score	81.11 ± 15.04
Items 1	4.26 ± 0.68
Items 2	4.20 ± 0.69
Items 3	4.22 ± 0.65
Items 4	4.30 ± 0.65

### 3.4 Correlations between spiritual climate and related variables among oncology nurses

The findings of *Pearson* correlation analysis showed that occupational coping self-efficacy, perceived organizational support, and inclusive leadership were all positively correlated with spiritual climate (*p* < 0.01), as detailed in Table 3.

**Table 3:** Results of the correlation analysis of the spiritual climate of oncology nurses.

Variable	1	2	3	4
1. SCS-C	1			
2. OCSE-N	0.609^**^	1		
3. ILS	0.650^**^	0.573^**^	1	
4. POS	0.659^**^	0.636^**^	0.682^**^	1

^**^p < 0.01.

### 3.5 Multiple linear regression analysis of spiritual climate

The method of independent variable assignment is shown in Table 4. The multiple linear regression results showed that the head nurse position (β = 0.099, *p* = 0.032), occupational coping self-efficacy (β = 0.242, *p* < 0.001), inclusive leadership (β = 0.319, *p* < 0.001) and perceived organizational support (β = 0.272, *p* < 0.001) were significantly associated with nurses' spiritual climate, making it statistically significant (*R*^2^= 0.553, adjusted *R*^2^ = 0.541, *F* = 46.847, *p* < 0.001), and explain 54.1% of the variance of the dependent variable (Table 5). Multicollinearity was assessed using variance inflation factors. VIF values for all predictors ranged from 1.209 to 2.298, well below the recommended threshold of 5, indicating that multicollinearity was not a concern.

**Table 4:** The method of independent variable assignment.

Independent variable	Assignment of values
Age	< 25 = 1; 25–35 = 2; 36–45 = 3; >45 = 4
Administrative position	No position (0, 0, 0); nursing team leader (0, 1, 0); head nurse (0, 0, 1)
Number of night shifts per month	0 = 1; 1–4 = 2; 5–9 = 3; >9 = 4
OCSE-N	Input as raw data
POS	Input as raw data
ILS	Input as raw data

**Table 5:** Multiple linear regression analysis of factors associated with spiritual climate of oncology nurses (*N* = 273).

Variable	*B*	*SE*	β	*t*	*p*	95% *CI*	Collinearity statistics	
							Tolerance	VIF
(Constant)	6.415	6.034		1.063	0.289			
Age	−1.283	0.900	−0.068	−1.425	0.155	−3.055, 0.490	0.738	1.355
Nursing team leader	1.515	2.004	0.034	0.756	0.450	−2.432, 5.461	0.827	1.209
Head nurse	4.993	2.312	0.099	2.159	**0.032**	0.440, 9.546	0.798	1.253
Number of night shifts per month	0.345	0.779	0.021	0.442	0.659	−1.189, 1.879	0.758	1.319
OCSE-N	0.681	0.158	0.242	4.305	**< 0.001**	0.370, 0.992	0.536	1.867
ILS	0.827	0.150	0.319	5.494	**< 0.001**	0.530, 1.123	0.500	2.000
POS	0.412	0.094	0.272	4.370	**< 0.001**	0.226, 0.598	0.435	2.298

*R*^2^ = 0.553, adjusted *R*^2^ = 0.541, *F* = 46.847, *p* < 0.001. Bold values indicate statistical significance (*p* < 0.05).

### 3.6 Robustness check

Regression assumptions were assessed using the central limit theorem and the Breusch–Pagan test. Given the sample size of 273, the central limit theorem ensures that the sampling distribution of regression coefficients approximates normality (Wooldridge, 2015), making the analysis robust to minor violations of the normality assumption. Homoscedasticity was examined using the Breusch–Pagan test, which indicated heteroscedasticity (*p* < 0.05).

To ensure robust statistical inference despite heteroscedasticity, we re-estimated the regression model using bootstrap resampling with 5,000 samples and bias-corrected and accelerated (BCa) confidence intervals. As shown in Table 6, the bootstrap regression results were consistent with the ordinary least squares estimates, confirming the robustness of the findings. Occupational coping self-efficacy (*B* = 0.681, BCa 95% *CI* [0.346, 1.019], *p* < 0.001), inclusive leadership (*B* = 0.827, BCa 95% *CI* [0.377, 1.218], *p* < 0.001), and perceived organizational support (*B* = 0.412, BCa 95% *CI* [0.137, 0.743], *p* = 0.004) were significant positive predictors of spiritual climate. Among administrative positions, head nurse was significantly associated with spiritual climate (*B* = 4.993, BCa 95% *CI* [0.699, 9.400], *p* = 0.030), while nursing team leader was not (*B* = 1.515, BCa 95% *CI* [−2.316, 5.567], *p* = 0.450). Age (*B* = −1.283, BCa 95% *CI* [−2.851, 0.283], *p* = 0.125) and monthly night shift frequency (*B* = 0.345, BCa 95% *CI* [−1.297, 1.977], *p* = 0.684) were not significant predictors.

**Table 6:** Bootstrap multiple regression analysis for predictors of spiritual climate.

Variable	*B*	Bootstrap *SE*	*p-*value	BCa 95% *CI*
(Constant)	6.415	6.953	0.366	[−7.764, 20.431]
Age	−1.283	0.834	0.125	[−2.851, 0.283]
Nursing team leader	1.515	1.997	0.450	[−2.316, 5.567]
Head nurse	4.993	2.279	**0.030**	[0.699, 9.400]
Number of night shifts per month	0.345	0.856	0.684	[−1.297, 1.977]
OCSE-N	0.681	0.181	**< 0.001**	[0.346, 1.019]
ILS	0.827	0.219	**< 0.001**	[0.377, 1.218]
POS	0.412	0.145	**0.004**	[0.137, 0.743]

Bootstrap results based on 5,000 samples. BCa, bias-corrected and accelerated confidence intervals. Bold coefficients are statistically significant (*p* < 0.05).

## 4 Discussion

### 4.1 Analysis of the current situation of spiritual climate among oncology nurses

The spiritual climate score among oncology nurses in this study was 81.11 ± 15.04, which was higher than scores reported in a study of Greek clinical nurses (Fradelos and Tzavella, 2020) and another study involving Chinese ICU nurses (Hu et al., 2025). This finding could be attributed to the variations in workplace environments across specialties. Research has demonstrated that different clinical settings can significantly impact healthcare professionals' work perceptions and well-being (Pierce et al., 2007). Oncology nurses work in specialized units where interdisciplinary collaboration and holistic care are emphasized, which may contribute to a more supportive spiritual climate. In contrast, ICUs are characterized by fast-paced working environments, high acuity of patient care, and technical complexities (Wang et al., 2024). In such settings, interactions are often brief and task-focused, which may offer fewer opportunities for the relational exchanges that support spiritual climate. Among the four entries of the spiritual climate scale, the highest scored by the oncology nurses in this study was entry 4 “I am in a department where everyone can express his or her ideas and we respect and understand each other”, which is consistent with the findings of (Xie et al. 2025). However, the lowest scored entry was entry 2 “Doctors and nurses respect the opinions and ideas that I put forward”, which differs from the result of (Cruz et al. 2018). This finding could be attributed to the differences in economic, historical, and cultural contexts across countries, which have shaped distinct ways of thinking and hospital management models. In the Chinese health care context, hierarchical dynamics in nurse-physician relationships may be more pronounced, which may contribute to nurses perceiving that their professional opinions receive less attention from physicians. Hospitals and administrators should maintain internal team support, establishing structured interprofessional communication platforms. Measures such as joint ward rounds and case discussions could help improve nurses' spiritual climate.

### 4.2 The results of *t*-test and ANOVA analysis

The results of *t*-test and ANOVA indicated that there were significant disparities in oncology nurses' spiritual climate scores across age, administrative positions, and night shift frequency. *Post hoc* comparisons showed that oncology nurses under 25 years old had significantly higher SCS scores than the 25–35 nurses (*p* = 0.023) and the over 45 nurses (*p* = 0.015). A possible explanation is that younger nurses, being in the early stages of their careers, may exhibit greater sensitivity to the perceived value of their work and may benefit from enhanced organizational support for self-expression during their standardized training period. Head nurses had significantly higher SCS scores than nurses without administrative position (*p* = 0.018). The professional growth and autonomy associated with career advancement allow nurses in higher-level positions to cultivate a greater sense of meaning in their work. Nurses working no night shifts monthly had significantly higher SCS scores than those working more than nine night shifts per month (*p* = 0.021). This may be related to the fact that nurses working no night shifts have more opportunities for daytime team interactions, which may strengthen their interpersonal connections and sense of belonging, thereby enhancing their perception of spiritual climate. Therefore, hospitals and nursing managers should provide career development programs, establish health management and flexible scheduling for those over 45, and optimize night shift arrangements through frequency control and enhanced team support to improve the spiritual climate perception across the nursing workforce.

### 4.3 Analysis of the influencing factors of spiritual climate among oncology nurses

The results of the multiple linear regression analyses revealed that head nurse, occupational coping self-efficacy, perceived organizational support, and inclusive leadership were significantly associated with the spiritual climate of oncology nurses. Compared to staff nurses, those holding the position of head nurse exhibited significantly higher spiritual climate scores (β = 0.099, *p* = 0.032). The finding is consistent with the results of previous studies (Zhao et al., 2021). Research has shown that positive team climate and supportive interpersonal relationships among nursing staff are associated with better psychological well-being and work outcomes (Lönnqvist et al., 2025). Head nurses, by virtue of their managerial roles, typically have greater autonomy and more opportunities for meaningful interpersonal connections with colleagues, which may contribute to their more positive perceptions of workplace spiritual climate.

In addition, occupational coping self-efficacy represents one of the key personal factors associated with the spiritual climate, corroborating Lin et al.'s view on occupational coping self-efficacy (Lin et al., 2024). This may be explained by self-efficacy equipping staff with capabilities and resources to improve their achievements and alleviate burnout caused by demanding work (Bernales-Turpo et al., 2022), which may in turn enhance their perception of a supportive and meaningful work environment. Therefore, nursing managers should invest in self-efficacy enhancement, providing resources and training to alleviate emotional exhaustion and enable a positive spiritual climate.

Perceived organizational support was a key environmental factor significantly associated with the spiritual climate among oncology nurses, in line with the findings of (Gillet et al. 2013). Perceived organizational support fulfills nurses' fundamental socioemotional needs for affiliation and emotional support (Larsman et al., 2024), which may in turn relate to positive interpersonal relationships among nursing staff. These relationships, including nurse-colleague and nurse-manager interactions, have been shown to significantly influence nurses' perceptions of their practice environment (Tlhako et al., 2025). Nursing managers should provide personalized career development support and establish open communication platforms to systematically build an organizational support system, thereby fostering a spiritual climate within nursing teams that emphasizes the pursuit of meaning, mutual respect, and emotional connection.

The results indicated that inclusive leadership was significantly associated with spiritual climate among oncology nurses. A possible explanation is that inclusive leadership, characterized by openness, accessibility, and availability, fosters interpersonal trust and mutual respect between leaders and team members. By cultivating an environment where staff can confidently share their thoughts and concerns without fear of negative consequences, inclusive leadership may help nurses—maintain positive psychological resources and develop adaptive responses to workplace challenges (Liu et al., 2024). This supportive relational context may facilitate nurses' capacity to find meaning and purpose in their work, thereby enhancing their perception of spiritual climate. Therefore, nursing managers should emphasize the development of inclusive leadership behaviors to foster a positive workplace spiritual climate.

## 5 Limitations

This study has several limitations. First, the sample was recruited only from Jiangsu Province, which may limit the generalizability of findings to oncology nurses in other regions. Future studies should expand the geographic scope. Second, convenience sampling may introduce selection bias. Although we recruited nurses from 43 hospitals across 13 cities, the sample may not fully represent all oncology nurses in China. Probability sampling is recommended in future research. Third, self-reported questionnaires may be subject to self-report bias, including social desirability bias. We attempted to mitigate this by embedding an attention-check item and ensuring anonymity, but bias cannot be completely eliminated. Fourth, although Harman's single-factor test was conducted, this method has limitations in detecting common method bias. Future studies should employ more advanced techniques, such as the unmeasured latent method factor approach or marker variables. Fifth, the inability to compare respondents with non-respondents may introduce non-response bias. Sixth, the cross-sectional design precludes causal inferences. Longitudinal or experimental studies are needed to establish causality. Despite these limitations, this study provides valuable data on the spiritual climate among oncology nurses in Jiangsu Province, which can inform the development of targeted interventions and organizational strategies to improve nurse well-being and job satisfaction.

## 6 Conclusions

This study aims to describe the current state of spiritual climate among oncology nurses and its potential associated factors. The mean spiritual climate score among oncology nurses in Jiangsu Province, China, was 81.11 ± 15.04. Multiple linear regression analysis showed that the head nurse, perceived organizational support, occupational coping self-efficacy, and inclusive leadership explained 54.1% of the total variance. Therefore, it is imperative for hospital administrators to develop targeted strategies that cultivate a supportive spiritual climate for oncology nurses. Such initiatives are essential for fostering a positive and open organizational culture, which will ultimately enhance nursing resilience and optimize the patient care experience.

## Data Availability

The original contributions presented in the study are included in the article/supplementary material, further inquiries can be directed to the corresponding author.

## References

[B1] Bernales-Turpo D. Quispe-Velasquez R. Flores-Ticona D. Saintila J. Ruiz Mamani P. G. Huancahuire-Vega S. . (2022). Burnout, professional self-efficacy, and life satisfaction as predictors of job performance in health care workers: the mediating role of work engagement. J. Prim. Care Community Health 13:21501319221101845. doi: 10.1177/21501319221101845 35603465 PMC9125607

[B2] Bray F. Laversanne M. Sung H. Ferlay J. Siegel R. L. Soerjomataram I. . (2024). Global cancer statistics 2022: GLOBOCAN estimates of incidence and mortality worldwide for 36 cancers in 185 countries. CA Cancer J. Clin. 74, 229–263. doi: 10.3322/caac.21834 38572751

[B3] Carmeli A. Roni R.-P. Ziv E. (2010). Inclusive leadership and employee involvement in creative tasks in the workplace: the mediating role of psychological safety. Creat. Res. J. 22, 250–260. doi: 10.1080/10400419.2010.504654

[B4] Chen C. C. Tu H. Y. (2021). The effect of digital game-based learning on learning motivation and performance under social cognitive theory and entrepreneurial thinking. Front. Psychol. 12:750711. doi: 10.3389/fpsyg.2021.750711 34975642 PMC8716945

[B5] Çinar Özbay S. Gelin D. Durmuş Sarikahya S. Boztepe H. (2025). Trauma-informed care in pediatric oncology nursing. Psychooncology 34:e70360. doi: 10.1002/pon.70360 41388704

[B6] Cruz J. P. Alquwez N. Albaqawi H. M. Alharbi S. M. Moreno-Lacalle R. C. (2018). Nurses' perceived spiritual climate of a hospital in Saudi Arabia. Int. Nurs. Rev. 65, 559–566. doi: 10.1111/inr.12481 30239998

[B7] Cruz J. P. Alquwez N. Balay-Odao E. (2022). Work engagement of nurses and the influence of spiritual climate of hospitals: a cross-sectional study. J. Nurs. Manag. 30, 279–287. doi: 10.1111/jonm.13492 34619805

[B8] Cruz J. P. Alquwez N. Mesde J. H. Almoghairi A. M. A. Altukhays A. I. Colet P. C. (2020). Spiritual climate in hospitals influences nurses' professional quality of life. J. Nurs. Manag. 28, 1589–1597. doi: 10.1111/jonm.13113 32743944

[B9] Doram K. Chadwick W. Bokovoy J. Profit J. Sexton J. D. Sexton J. B. (2017). Got spirit? The spiritual climate scale, psychometric properties, benchmarking data and future directions. BMC Health Serv. Res. 17:132. doi: 10.1186/s12913-017-2050-5 28189142 PMC5303307

[B10] Faul F. Erdfelder E. Buchner A. Lang A. G. (2009). Statistical power analyses using G^*^Power 3.1: tests for correlation and regression analyses. Behav. Res. Methods 41, 1149–1160. doi: 10.3758/BRM.41.4.1149 19897823

[B11] Fradelos E. C. Tzavella F. (2020). Spiritual climate as is perceived by Greek clinical nurses. A validation study. Mater. Sociomed. 32, 66–70. doi: 10.5455/msm.2020.32.66-70 32410895 PMC7219727

[B12] Gillet N. Colombat P. Michinov E. Pronost A. M. Fouquereau E. (2013). Procedural justice, supervisor autonomy support, work satisfaction, organizational identification and job performance: the mediating role of need satisfaction and perceived organizational support. J. Adv. Nurs. 69, 2560–2571. doi: 10.1111/jan.12144 23551132

[B13] Guo J. B. Li Y. H. Zhang E. X. Li G. Q. (2023). Research progress on nurses' workplace spiritual climate. Chin. Nurs. Manag. 23, 595–599. doi: 10.3969/j.issn.1672-1756.2023.04.022

[B14] Hu L. T. Bentler P. M. (1999). Cutoff criteria for fit indexes in covariance structure analysis: conventional criteria versus new alternatives. Struct. Equ. Model. Multidiscip. J. 6, 1–55. doi: 10.1080/10705519909540118

[B15] Hu M. Wang Y. Zhang H. Wu C. Liang X. Zhang Y. . (2025). The relationship between spiritual climate and secondary traumatic stress in ICU nurses: the mediating role of moral resilience. Intensive Crit. Care Nurs. 87:103815. doi: 10.1016/j.iccn.2024.103815 39642416

[B16] Huang H. Zhang X. Tu L. Peng W. Wang D. Chong H. . (2025). Inclusive leadership, self-efficacy, organization-based self-esteem, and intensive care nurses' job performance: a cross-sectional study using structural equation modeling. Intensive Crit. Care Nurs. 87:103880. doi: 10.1016/j.iccn.2024.103880 39500700

[B17] Huang X. Y. Guo L. P. Zhang H. Y. (2021). Correlation between perceived organizational support and spiritual climate among operating room nurses in public hospitals. Evid. Based Nurs. 7, 1962–1966. doi: 10.12102/j.issn.2095-8668.2021.14.021

[B18] Ílvia Caldeira S. Timmins F. (2017). Implementing spiritual care interventions. Nurs. Stand. 31, 54–60. doi: 10.7748/ns.2017.e10313 28421952

[B19] Kudubeş A. A. Semerci R. Özbay S. Ay A. Boztepe H. (2023). Development and psychometric analysis of a pediatric oncology nurses' educational needs scale. Pediatr. Blood Cancer 70:e30285. doi: 10.1002/pbc.30285 36881503

[B20] Larsman P. Pousette A. Törner M. (2024). The impact of a climate of perceived organizational support on nurses' well-being and healthcare-unit performance: a longitudinal questionnaire study. J. Adv. Nurs. 80, 4921–4932. doi: 10.1111/jan.16188 38591844

[B21] Lin Q. Fu M. Sun K. Liu L. Chen P. Li L. . (2024). The mediating role of perceived social support on the relationship between lack of occupational coping self-efficacy and implicit absenteeism among intensive care unit nurses: a multicenter cross-sectional study. BMC Health Serv. Res. 24:653. doi: 10.1186/s12913-024-11084-y 38773420 PMC11110179

[B22] Liu L. Q. Li M. Q. Cao X. X. Zhou M. J. (2024). The moderating role of inclusive leadership between nurse conflict and nursing team work climate. Chin. Nurs. Res. 38, 2514–2518. doi: 10.12102/j.issn.1009-6493.2024.14.011

[B23] Lönnqvist K. Sinervo T. Kaihlanen A. M. Elovainio M. (2025). Psychosocial work characteristic profiles and health outcomes in registered nurses at different stages of their careers: a cross-sectional study. BMC Health Serv. Res. 25:214. doi: 10.1186/s12913-024-12164-9 39915838 PMC11800416

[B24] Mei A. Y. Han B. R. (2021). Research progress on occupational coping self-efficacy among nurses. Chin. J. Mod. Nurs. 27, 552–556. doi: 10.3760/cma.j.cn115682-20200603-03722

[B25] Pierce B. Dougherty E. Panzarella T. Le L. W. Rodin G. Zimmermann C. (2007). Staff stress, work satisfaction, and death attitudes on an oncology palliative care unit, and on a medical and radiation oncology inpatient unit. J. Palliat. Care 23, 32–39. doi: 10.1177/082585970702300105 17444460

[B26] Rhoades L. Eisenberger R. (2002). Perceived organizational support: a review of the literature. J. Appl. Psychol. 87, 698–714. doi: 10.1037/0021-9010.87.4.698 12184574

[B27] Shanafelt T. D. (2009). Enhancing meaning in work: a prescription for preventing physician burnout and promoting patient-centered care. JAMA 302, 1338–1340. doi: 10.1001/jama.2009.1385 19773573

[B28] Tlhako N. Coetzee S. K. Ajanaku O. J. Fourie E. (2025). The impact of workplace relationships on nurse-reported quality of care and patient safety in the North West Province. PLoS ONE 20:e0323620. doi: 10.1371/journal.pone.0323620 40397912 PMC12094723

[B29] Wang L. Chen Y. Yu H. Wu L. You A. Zheng X. . (2024). The experiences of newly qualified nurses in intensive care unit: a qualitative meta-synthesis. Front. Med. (Lausanne) 11:1458845. doi: 10.3389/fmed.2024.1458845 39540044 PMC11557346

[B30] Wang X. Xia Y. Gou L. Wen X. (2024). Exploring the influence of the spiritual climate on psychological empowerment among nurses in China: a cross-sectional study. BMC Nurs. 23:374. doi: 10.1186/s12912-024-02011-x 38831307 PMC11145847

[B31] Wooldridge J. M. (2015). Introductory Econometrics: A Modern Approach, 6th Edn. Boston, MA: Cengage Learning.

[B32] Wu X. X. Zhang Y. Wu J. F. Wan X. J. Hu Y. Liu Y. B. . (2019). Reliability and validity of the Chinese version of the spiritual climate scale—short form. Chin. Nurs. Res. 33, 2396–2399. doi: 10.12102/j.issn.1009-6493.2019.14.006

[B33] Xie B. Wu X. X. Bi Y. X. He Y. Y. Zhang Y. (2025). Status and influencing factors of spiritual climate among nurses in tertiary traditional Chinese medicine hospitals. Evid. Based Nurs. 11, 303–308. doi: 10.12102/j.issn.2095-8668.2025.02.021

[B34] Xu C. C. (2024). Classification and influencing factors of spiritual climate among oncology nurses based on latent profile analysis. Chin. Nurs. Res. 38, 3254–3259. doi: 10.12102/j.issn.1009-6493.2024.18.008

[B35] Zhai Y. X. Chai X. Y. Liu K. Meng L. D. (2021). Translation and psychometric testing of the Chinese version of the occupational coping self-efficacy scale for nurses. Mod. Prev. Med. 48, 423–426. doi: 10.20043/j.cnki.mpm.2021.03.010

[B36] Zhao Y. Wu S. D. Wan Y. K. (2021). Investigation on the perception of spiritual climate in the work environment among operating room nurses in tertiary hospitals. J. Clin. Nurs. 20, 69–72. doi: 10.3969/j.issn.1671-8933.2021.04.024

[B37] Zuo H. M. Yang H. (2009). Research on the relationship between perceived organizational support and organizational commitment among nurses. Chin. Nurs. Res. 23, 1341–1343. doi: 10.3969/j.issn.1009-6493.2009.15.013

