# Supplementary material for: Status and influencing factors of spiritual climate among oncology nurses: a cross-sectional study
Source: Front Psychol. 2026 May 14;17:1759724. doi: 10.3389/fpsyg.2026.1759724 (PMC13216816; doi:10.3389/fpsyg.2026.1759724)
Supplement: Supplementary file 1 [file Table_1.docx]

Supplementary Materials

Table 1. Model AVE and CR Results

| Factor | AVE | CR |
| --- | --- | --- |
| 1. SCS-C | 0.754 | 0.925 |
| 2. OCSE-N | 0.551 | 0.916 |
| 3. ILS | 0.780 | 0.970 |

**Table 2. Pearson correlations and square roots of AVE**

| **Variable** | **1** | **2** | **3** | **4** |
| --- | --- | --- | --- | --- |
| 1. SCS-C | **0.868** |  |  |  |
| 2. OCSE-N | 0.609** | **0.742** |  |  |
| 3. ILS | 0.650** | 0.573** | **0.883** |  |
| 4. POS | 0.659** | 0.636** | 0.682** | **0.864** |
